# Syngas enhancement for Fischer-Tropsch integration via solid oxide electrolyzer cell co-electrolysis with or without methane

**DOI:** 10.1016/j.isci.2024.111014

**Published:** 2024-09-23

**Authors:** Marina Machado, Ricardo Lopes de Souza Junior, João Monnerat Araújo Ribeiro de Almeida, Pedro Nothaft Romano, Marco Aurélio Suller Garcia

**Affiliations:** 1 Instituto SENAI de Inovação em Biomassa - ISI Biomassa, SENAI-MS, Av Angelina Tebet, 777, Três Lagoas 79640-250, MS, Brazil; 2 Instituto de Química, Universidade Federal do Rio de Janeiro (UFRJ), Rio de Janeiro 21941-909, RJ, Brazil; 3 LIPCAT (Laboratório de Intensificação de Processos e Catálise), Universidade Federal do Rio de Janeiro (UFRJ), Rio de Janeiro 21941-594, RJ, Brazil; 4 Campus Duque de Caxias, Universidade Federal do Rio de Janeiro (UFRJ), Rio de Janeiro 25245-390, Brazil; 5 Nanotechnology Engineering Program, Alberto Luiz Coimbra Institute for Graduate Studies and Research in Engineering, COPPE, Universidade Federal do Rio de Janeiro (UFRJ), Rio de Janeiro 21941-972, RJ, Brazil; 6 Department of Chemistry, Federal University of Maranhão (UFMA), São Luís 65080-805, MA, Brazil

**Keywords:** Electrochemistry, Applied sciences

## Abstract

The transition toward a sustainable energy framework requires developing innovative methods for fuel generation that utilize renewable resources and decrease carbon footprints. Thus, the review overviews current advancements in solid oxide electrolysis cell (SOEC) technology, specifically focusing on its application in co-electrolysis processes to produce syngas with different H_2_:CO ratios, essential for Fischer–Tropsch synthesis. It emphasizes the potential of integrating partial methane oxidation reaction into the electrolysis process. By examining recent developments in electrode, electrolyte materials, and system design, the review highlights how these technological enhancements can reduce energy consumption, improve system durability, and facilitate the integration of renewable energy sources. Additionally, the role of methane assistance in SOECs is discussed, illustrating its impact on operational efficiency. Then, future research directions that could optimize syngas production and expand the applicability of SOEC technology in industrial settings are proposed, supporting the transition to a more sustainable energy landscape.

## Graphical abstract

**Figure:**
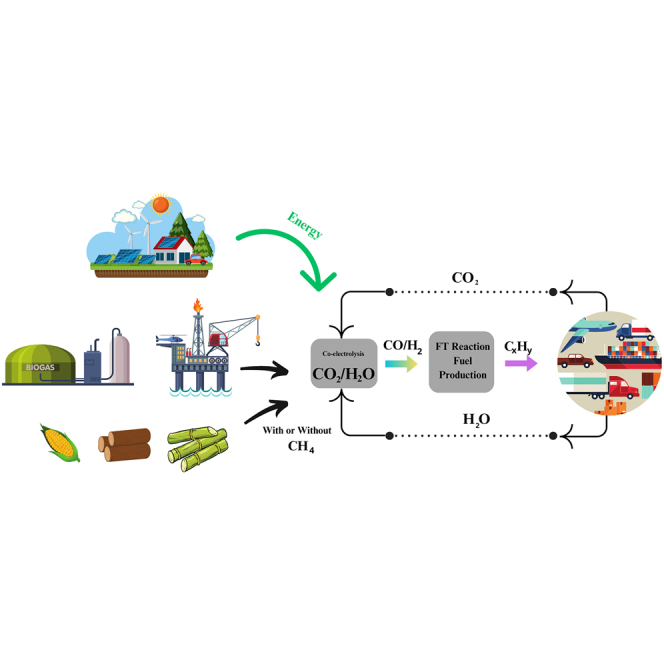

## Introduction

As global economies expand and populations rise, energy demand is projected to grow significantly in the coming years. Regrettably, this increased need is mostly fulfilled by fossil fuels, which have caused substantial environmental harm. The world’s reliance on these energy sources accelerates pollution, contributes to climate change, and poses severe risks to biodiversity and human health. Consequently, efforts are directed at transitioning the current energetic matrix into clean and renewable energy sources.^1^^,^^2^^,^^3^ In this context, it becomes evident that when implemented effectively, renewable energy sources not only offer solutions to energy issues but also have the potential to catalyze social and economic progress while improving energy accessibility.^4^

Facing the challenges of the transition, the Intergovernmental Panel on Climate Change (IPCC) advised that the world must reach net-zero CO_2_ emissions by 2050 to contain the global temperature increase to 1.5°C.^5^ However, in light of recent projections, it is clear that accelerated actions are required. In addition, COP28, held in Dubai, emphasized the critical role of methane (CH_4_) reduction by 2030, with new commitments aimed at cutting emissions across various sectors. This attempt is expected to reduce global greenhouse gas emissions substantially.^6^^,^^7^ Thus, using CH_4_ in industrial settings aligns effectively with the initiative. Furthermore, converting carbon dioxide (CO_2_) into valuable products or energy sources is imperative and highly sought after.^8^^,^^9^^,^^10^^,^^11^

**Figure 1:**
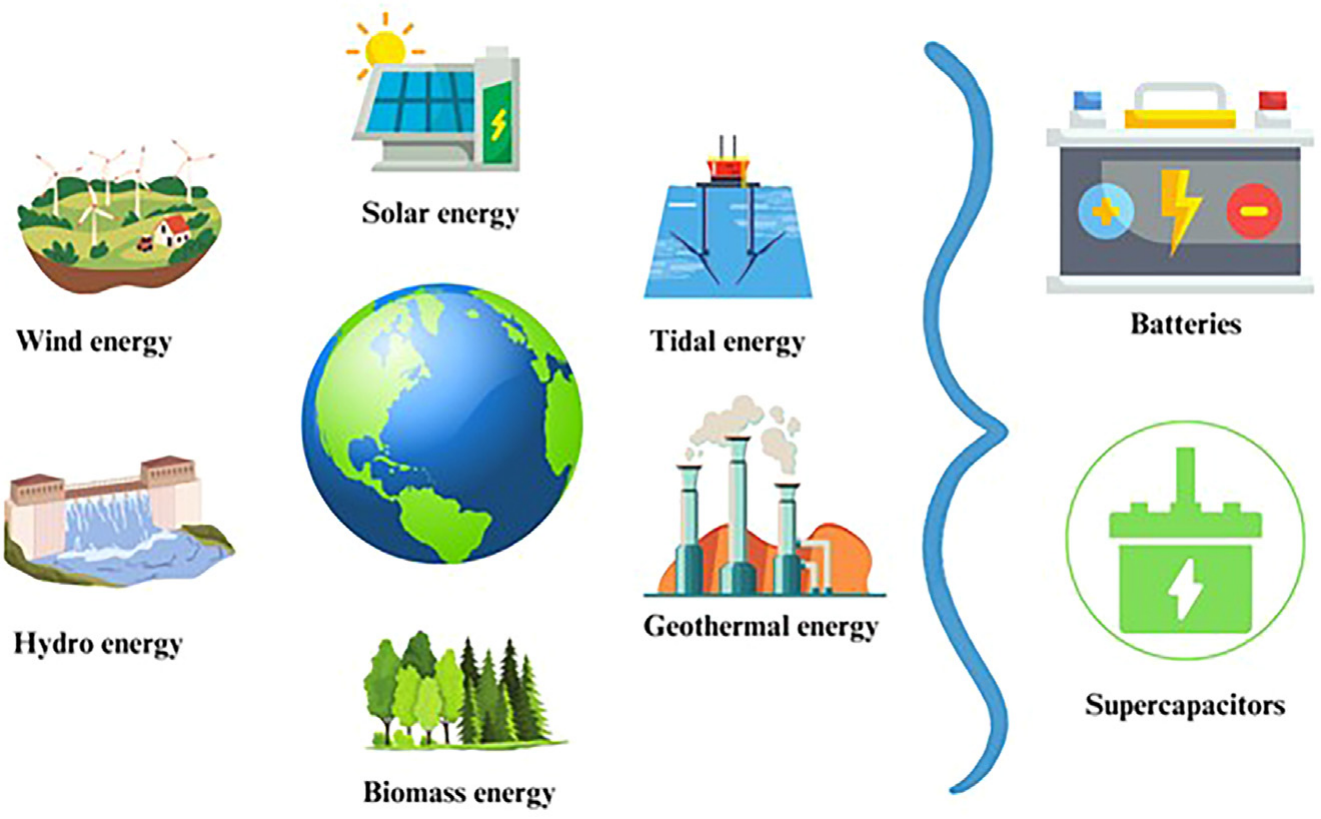
Different renewable energy resources and storage devices

Wind, hydropower, and solar energy are the most prevalent renewable resources with promising potential for application in chemical processes beyond their conventional energy production roles. Additionally, tidal, geothermal, and biomass sources are undergoing extensive research.^12^^,^^13^ However, the intermittent nature of wind, solar, and other renewable sources challenges the need for a consistent power supply.^14^ Batteries and supercapacitors offer solutions by storing excess energy and enhancing grid stability (Figure 1)^15^^,^^16^^,^^17^^,^^18^^,^^19^^,^^20^^,^^21^^,^^22^ but they face limitations in solving grid integration problems due to their finite energy storage capacity, degradation over time, and the high costs associated with large-scale deployment, among others.^23^^,^^24^

In this scenario, electrolytic cell technologies have emerged as promising solutions due to their high efficiency, environmental sustainability, and versatile applications. Various types of electrolysis cells have been developed, including alkaline electrolysis, proton exchange membrane (PEM), and solid oxide electrolysis cells (herein referred to as SOECs).^25^^,^^26^^,^^27^ Each cell type possesses distinct features, including operational temperature, electrolyte type, electrode materials, charge carriers, efficiency, lifetime, energy consumption, and costs. As such, the development and refinement of these technologies continue to play a crucial role in the transition to sustainable energy solutions.

Alkaline electrolysis cells operate at temperatures below 100°C, utilizing a liquid solution as the electrolyte and a metal as the cathode, making them suitable for environments with lower thermal energy; also, they offer an efficiency range of 60–75% and the highest expected lifetime among the three types of cells at 80,000 h. However, they exhibit a high energy consumption and suffer from low current density, poor energy efficiency, and insufficient stability, although they benefit from rich product outputs and high Faradaic efficiency.^28^ In contrast, PEM cells operate at slightly higher temperatures and utilize a polymer electrolyte, metal cathodes, and more expensive anode materials such as Pt or Ir/Pt. Their design enables a higher efficiency range of 67–82% and a considerable lifetime of 60,000 h, although they have a slightly higher energy consumption. Their advantages include high current density and Faradaic efficiency, but like alkaline electrolysis cells, they struggle with poor energy efficiency and insufficient stability.^28^^,^^29^^,^^30^^,^^31^

**Figure 2:**
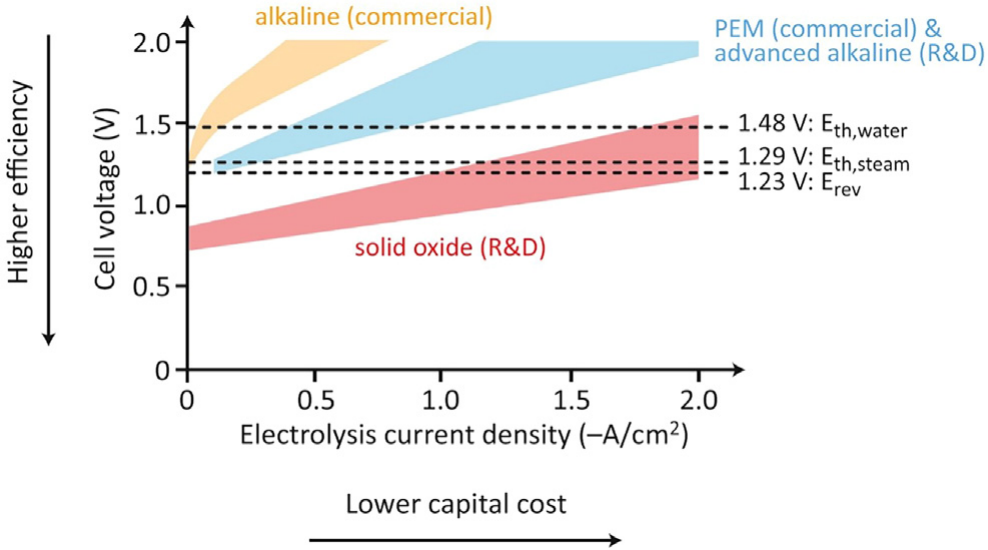
Polarization curves for three types of electrolyzers exhibiting different ranges “E_th_ water” and “E_th_ steam” represent the thermoneutral voltages for water and steam, respectively, while “E_rev_” denotes the reversible potential for water electrolysis. Reproduced with permission from ref.^32^; copyright 2011, Elsevier.

SOECs operate at a significantly higher temperature (>600°C) and utilize a ceramic electrolyte, perovskite, and cermet/perovskite for cathode and anode materials. Such configuration supports their high-efficiency range of 85–100% but at the cost of the shortest expected lifetime at 40,000 h. However, SOECs’ high operational temperatures contribute to their high energy efficiency and good stability. Furthermore, high-temperature cells typically exhibit lower internal resistance than low-temperature cells. Even with equivalent catalytic activity, higher reaction rates are attained in high-temperature cells due to their greater thermal activation. The polarization curves in Figure 2 illustrate a comparison among the discussed systems.^32^ This graph shows the possibility of choosing the efficiency as the electrolysis operates. High-temperature cells typically exhibit lower internal resistance than low-temperature cells, even when the catalytic activity is equivalent since the reactions in high-temperature cells are more extensively thermally activated, leading to higher reaction rates.

While exploring alternative energy generation systems is important, producing syngas (H_2_:CO) for several industrial applications while mitigating CO_2_ quantity in the atmosphere is crucial. Consequently, in recent decades, extensive efforts have been directed toward technologies for CO_2_ sequestration integrated with its conversion to products with added value.^33^^,^^34^^,^^35^ The CO_2_:H_2_O co-electrolysis emerged as a promising approach, particularly when using electricity from renewable and clean energy sources.^36^^,^^37^ In this context, it is worth noting that conducting co-electrolysis in SOECs at high temperatures enables gas-phase reactions to control thermodynamics and kinetics, thus reducing operating costs and improving efficiency.^38^

**Figure 3:**
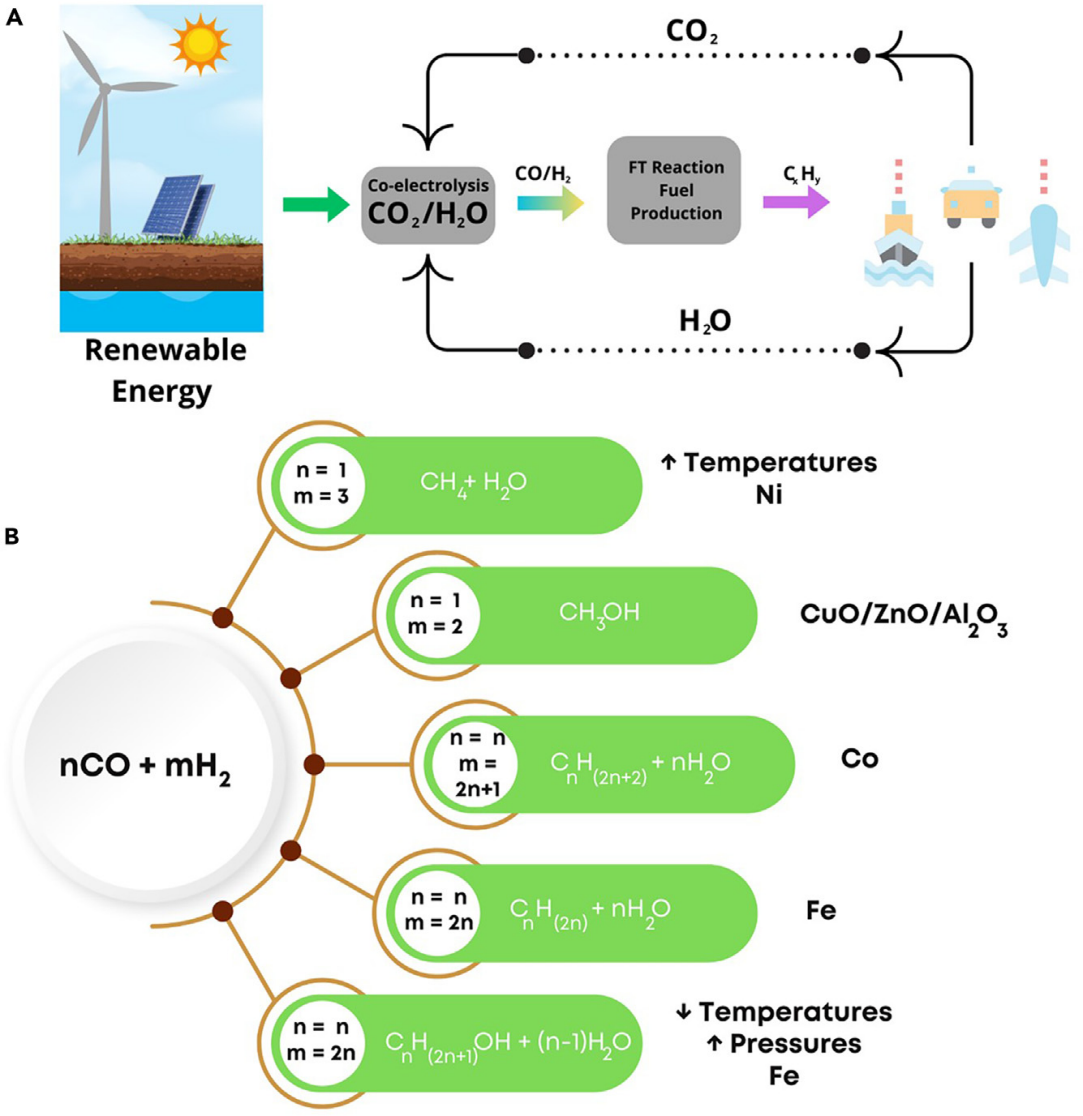
Integration of renewable energy with FT synthesis (A) Sustainable carbon-recycling strategy with H_2_O:CO_2_ co-electrolysis in SOECs. (B) FT synthesis based on different H_2_:CO ratios and catalysts.

Interestingly, the co-electrolysis SOECs can be directly integrated with the Fischer–Tropsch (FT) synthesis, enhancing the conversion efficiency of syngas into various hydrocarbon fuels, thus offering a promising pathway to reduce carbon footprint and promote energy sustainability.^39^^,^^40^ Furthermore, employing such a method would contribute to embracing a carbon-recycling strategy (Figure 3A). In contrast to conventional methods such as coal gasification or steam reforming of natural gas for syngas production, the solid oxide co-electrolysis process operates without the consumption of fossil fuels.^41^^,^^42^^,^^43^ Moreover, it facilitates the electrochemical utilization of CO_2_, thereby mitigating emissions rather than contributing to them.^44^^,^^45^ However, while the technique holds considerable promise in addressing energy and environmental concerns, numerous challenges must be managed before achieving practical feasibility. Thus, much effort is still needed, and some achievements will be addressed herein.

In this context, for the production of fuels and chemicals, the composition of syngas is critical and varies based on the intended application.^46^ For instance, synthesizing methanol requires an H_2_:CO ratio of 2:1. Meanwhile, the FT synthesis, which converts syngas into liquid hydrocarbons, operates efficiently within a wider H_2_:CO ratio range of 0.3–4, accepting various product specifications and process conditions (Figure 3B).^47^^,^^48^ Thus, integrating SOECs with FT processes is crucial for enhancing carbon efficiency in conversion to liquid fuels. However, for perfect integration, the H_2_:CO ratio should be adjusted for the intended application, avoiding the need for an energy-intensive gas shift reaction. Such integration would boost carbon efficiency and align with sustainable energy goals.

The study of fuel-assisted co-electrolysis in an SOEC has gained significant attention recently.^49^^,^^50^^,^^51^ Such a method is an advanced electrolysis system that uses fuel to enhance the electrolysis process; such fuel could be NO_x_ or CH_4_, although the latter is more commonly used.^50^^,^^52^^,^^53^ In this setup, the presence of fuel helps by reducing the overall electrical energy required to drive the electrolysis, thereby improving efficiency.^52^^,^^54^^,^^55^ Additionally, reactions such as the partial oxidation of methane (POM) can occur, significantly enhancing syngas production. The methane-assisted SOEC co-electrolysis process is complex, involving various reactions and species. On the electrodes, reactions are influenced by gas composition, flow rate, temperature, and current, requiring adjustments to obtain a high-rate syngas production with an ideal H_2_:CO ratio.^52^ Challenges when operating the cell include carbon deposition, which affects temperature and steam/carbon ratio and leads to cell degradation. By optimizing conditions and enhancing catalyst efficiency, the carbon build-up can be suppressed, thus enabling the production of high-quality syngas with the desired H_2_:CO ratios.

Given the context, we will summarize the latest advancements in SOEC H_2_O:CO_2_ co-electrolysis systems, including their fundamental mechanisms and the key electrodes and electrolytes utilized. Then, different strategies to obtain tailored H_2_:CO ratios will be addressed, including examining operational parameters and catalysts' compositions. The discussion will extend to the integration with CH_4_-assisted processes, especially POM reactions, demonstrating the minimization of overpotential while optimizing the CO:H_2_ ratio for FT reaction integration. Additionally, we will explore CH_4_-assisted processes that are still in the developmental phase, based on simulations, to shed light on their potential applications. The review will also overview degradation mechanisms and some mitigation strategies. The focus will be on emerging trends and future research requirements for integrated SOECs, emphasizing the critical transition from reliance on fossil fuels to adopting clean and renewable energy sources.

## SOEC technology for H_2_O:CO_2_ Co-Electrolysis

### Fundamentals and thermodynamics of high-temperature H_2_O:CO_2_ co-electrolysis

**Figure 4:**
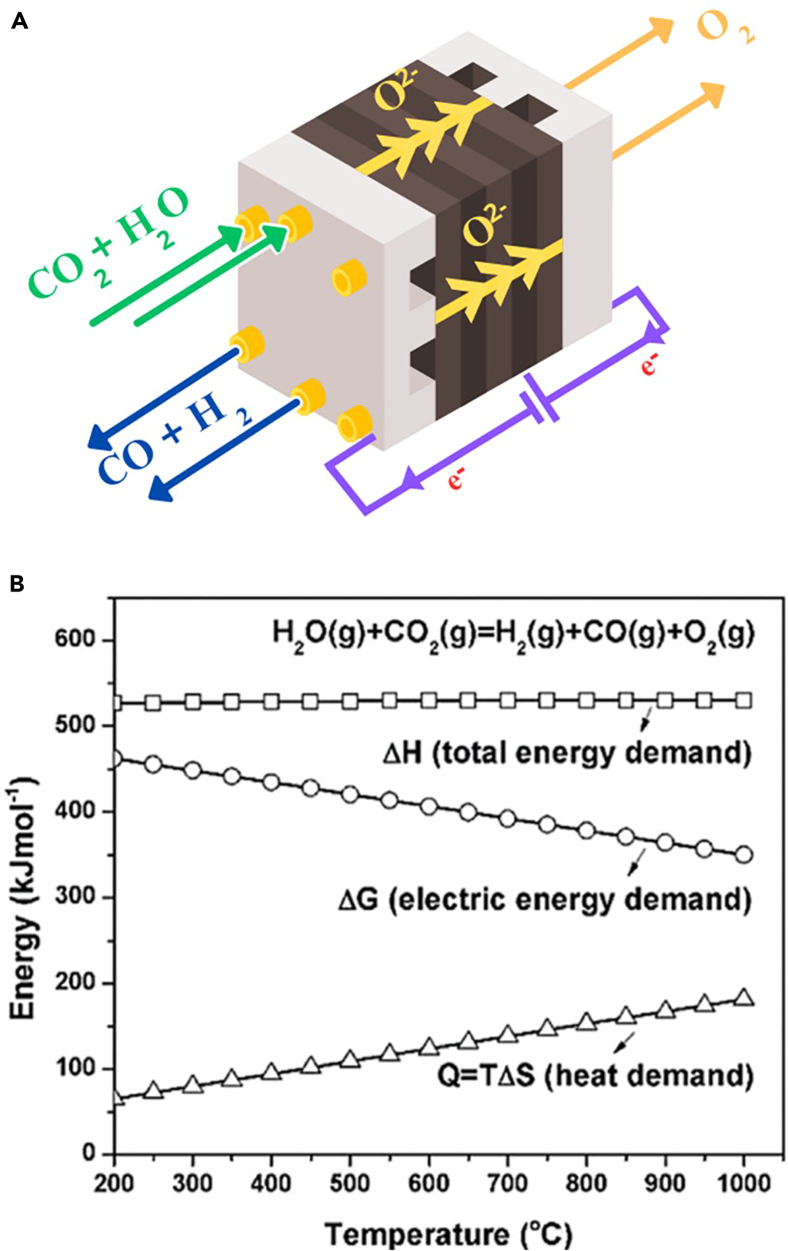
Characteristics of the co-electrolysis cells (A) Diagram illustrating the co-electrolysis of CO_2_ and H_2_O utilizing an SOEC to produce syngas and oxygen. (B) Relationship among the enthalpy change (ΔH), Gibbs free energy change (ΔG), and entropy change (ΔS) in electrolysis reactions involving H_2_O:CO_2_. Adapted with permission from ref.^56^; copyright 2015, Elsevier.

The high-temperature H_2_O:CO_2_ co-electrolysis (>600°C) is based on an oxide ion (O^2−^) conducting electrolyte and high operating temperatures, which are necessary for reducing the ohmic resistance of the electrolyte [56]. The electrolyzer consists of a cathode (a fuel electrode), an anode (an oxygen electrode), and a dense electrolyte (an ionic conductor). CO_2_ and H_2_O are supplied to the cathode, where electrons dissociate by applying an external electricity source into CO, H_2_, and O^2−^. During the device’s operation, the O^2−^ ions migrate across the dense electrolyte from the cathode to the anode, releasing oxygen at the anode and producing syngas at the cathode (Figure 4A). This process can be represented by Reactions (1), (2), and (3)."

(Equation 1) $$\text{Cathode}:{\text{CO}}_{2\left(g\right)}+2e^{-}\;⇌\;{\text{CO}}_{\left(g\right)}+H_{2}O_{\left(g\right)}$$

(Equation 2) $$H_{2}O_{\left(g\right)}+2e^{-}\;⇌\;H_{2\left(g\right)}+O^{2-}$$

(Equation 3) $$\text{Anode}:2\;O^{2-}\;⇌\;O_{2\backslash \left(g\right)}+4e^{-}$$

CO_2_ can be captured from power plants or industrial emissions, and the resulting syngas can be converted into synfuel, effectively transforming waste into usable fuel. Such a process will be further discussed in systems integrated with FT reactions. Moreover, using clean energy to power this process aligns with the current goal of transitioning to renewable energy. In addition to the electrolysis reaction mentioned earlier, the reverse water gas shift (RWGS) reaction described in Reaction (4) also occurs during high-temperature co-electrolysis processes.

(Equation 4) $${\text{CO}}_{2\left(g\right)}+H_{2\left(g\right)}\;⇌\;{\text{CO}}_{\left(g\right)}+H_{2}O_{\left(g\right)}$$

The interrelations between the enthalpy change (ΔH), Gibbs free energy change (ΔG), and entropy change (ΔS) for co-electrolysis reactions involving CO_2_ and H_2_O are shown in Figure 4B. As depicted, ΔH, ΔG, and TΔS represent the total energy demand, the electrical energy demand, and the heat demand, respectively. Despite the temperature increase, the total energy requirement remains constant. The rise in heat demand is correlated with a decrease in electrical energy demand for both H_2_O and CO_2_ splitting, attributed to a positive entropy change (ΔS > 0).^56^ High-temperature electrolysis, compared to low or intermediate-temperature operations, can efficiently use both electricity and heat, achieving nearly 100% efficiency in electricity-to-syngas conversion and promoting high reaction rates. Consequently, this leads to diminished internal cell resistance and enhanced syngas productivity at the same voltage.

Therefore, from a thermodynamic perspective, high-temperature operation is preferred for both H_2_O and CO_2_ electrolysis in terms of efficiency and cost-effectiveness. High-temperature H_2_O:CO_2_ co-electrolysis using SOECs shows promising potential for future cost reductions and efficiency enhancements, as it boasts higher energy efficiency compared to separate electrolysis processes of H_2_O and CO_2_.^57^ Its advantages are attributed to lower energy consumption, fewer electrolysis steps, and the requirement for only one reactor to operate.^45^^,^^58^

### Co-electrolysis materials in SOECs

This section overviews key materials used in SOEC systems for H_2_O:CO_2_ co-electrolysis. It highlights the significant contributions of these materials and is structured to simplify understanding through subsequent sections. Interestingly, these materials are used not only for SOECs but also for all Solid Oxide (Fuel and Electrolysis) Cells (SOCs) technologies. The main difference between Solid Oxide Fuel Cells (SOFCs) and SOECs lies in their operational requirements due to the reverse nature of their functions. SOFCs generate electricity through fuel oxidation, while SOECs utilize electricity to split water and CO_2_ into syngas or oxygen.^59^ Thus, both systems can employ similar materials for electrodes and electrolytes. However, the performance requirements may lead to different material choices or modifications to optimize fuel oxidation and oxygen reduction in SOFCs and electrolysis and gas production in SOECs. These optimizations focus on durability, ion conductivity, electronic conductivity, and catalytic activity under their respective operating conditions.

#### Electrolyte materials

The electrolytes in SOECs must exhibit high ionic conductivity, negligible electron conductivity, stability under operating conditions (high temperatures and reactive environments), and compatibility with other cell components to prevent degradation and ensure long-term operation.^60^ The electrolyte is often the primary factor influencing a cell’s ohmic resistance; therefore, the material’s ability to effectively conduct oxygen ions at high temperatures is crucial for the electrochemical conversion processes in SOECs. In this context, Yttria-stabilized zirconia (YSZ) is the most widely used electrolyte material due to its excellent oxygen ion conductivity and stability.^61^ It is typically used at high temperatures ranging from 700°C to 1000°C, which can lead to issues such as coarsening, segregation, and delamination in other components of the system; thus, to minimize the ohmic resistance associated with the electrolyte, the reduction in YSZ thickness to less than 20 μm is advisable.^62^ Additionally, various doping strategies have been extensively explored to enhance the ionic conductivity of YSZ.^63^^,^^64^^,^^65^

Scandium-stabilized zirconia (ScSZ) is a notable alternative, offering superior oxygen ion conductivity.^66^ To further boost the low-temperature performance of ScSZ, doping with a small amount of cerium has proven effective. However, this improvement comes at the cost of reduced mechanical strength and structural stability.^67^ Other combinations, such as samarium-doped ceria (SDC), have also been extensively studied. SDC, composed of cerium dioxide doped with samarium oxide, offers good ionic conductivity at intermediate temperatures, making it a preferred choice in systems operating between 500°C and 700°C. Also, lanthanum gallate-based electrolytes (LSGM), a lanthanum gallium oxide doped with strontium and magnesium, is known for its high ionic conductivity and good chemical stability, making it suitable for high-performance SOEC designs.^68^^,^^69^ Additionally, doped barium cerate, such as BaCe_0.8_Y_0.2_O_3-δ_ (BCY), and doped barium zirconate, like BaZr_0.8_Y_0.2_O_3-δ_ (BZY), are also used due to their protonic conductivity, which can be advantageous in certain SOEC applications.^70^^,^^71^

Another promising electrolyte candidate is the perovskite LaGaO_3_ doped with Sr and Mg, specifically La_0.9_Sr_0.1_Ga_0.8_Mg_0.2_O_3−δ_ (LSGM). This material demonstrates excellent oxygen ion conductivity at intermediate temperatures and maintains high ionic conduction across a broad range of oxygen partial pressures.^72^^,^^73^ Interestingly, LSGM’s conductivity surpasses that of YSZ and is on par with doped ceria. Unlike doped ceria, LSGM lacks reducible cations, meaning it does not exhibit electronic conduction at low oxygen partial pressures. Other oxide ion conductors include bismuth oxide-based materials, such as yttria-stabilized bismuth oxide (YSB) and erbia-stabilized bismuth oxide (ESB)^74^^,^^75^; apart from these examples, the literature brings several options.^59^^,^^76^^,^^77^

#### Electrode materials

Important electrode features for SOECs include high electrical conductivity, stability at operational temperatures, catalytic activity for the desired electrochemical reactions, durability under cycling conditions, and compatibility with the electrolyte to minimize degradation.^78^^,^^79^ In SOECs, there are two key electrodes: the fuel electrode (also known as the cathode) and the oxygen electrode (also known as the anode). These electrodes serve different purposes and are made from various materials to optimize their performance for their respective reactions.

There has been a notable shift from using traditional noble metals, like platinum, to adopting perovskite-based materials for oxygen electrodes. Such a change is driven by the perovskites' affordability and their enhanced compatibility with the electrolyte systems in use.^80^^,^^81^ Perovskites, particularly with the typical ABO_3_ structure in compounds like SrTiO_3_, are favored because of their structural setup. Here, larger A-site cations—often rare-earth or alkaline-earth ions—pair with smaller B-site transition metals in octahedral coordination, providing the perovskites with a range of valuable properties such as robust ionic conductivity and potential ferromagnetism, both critical for their functionality in SOECs.^82^ Specific perovskites such as lanthanum manganite (LaMnO_3_) and lanthanum strontium manganite (La_1−x_Sr_x_MnO_3−δ_ - LSM) stand out due to their exemplary performance in SOEC settings. Incorporating Sr^2+^ in place of La^3+^ induces p-type conductivity and considerably increases electronic conductivity, reaching between 200 and 490 S/cm at 1000°C when x is 0.5.^83^ Such a strategic substitution boosts the oxygen diffusion and surface exchange coefficients, enhancing the cell’s efficiency in oxygen evolution reactions (OER).

Moreover, researchers have engineered composite oxygen electrodes to maximize these properties by integrating perovskite oxides with YSZ. This combination effectively expands the triple-phase boundaries (TPB), significantly boosting the electrochemical activity. These composites are carefully designed to synergize the LSM’s robust electrical conductivity with YSZ’s ionic conductivity, carefully adjusting stoichiometry and microstructure for optimal performance.^84^^,^^85^ However, challenges persist, especially in maintaining performance at the high temperatures that SOEC operations often require. Such conditions can lead to the rapid deterioration of nanostructured elements, thereby reducing their functional capacity. This issue has guided ongoing research toward developing perovskites with mixed ionic and electronic conducting (MIEC) properties, which are more stable and perform better under the demanding conditions of SOEC operations.^86^ Widely used MIECs include lanthanum strontium cobaltite (LSC) and lanthanum strontium cobaltite-ferrite (LSCF), which possess mixed ionic and electronic conductivity.^78^ However, the primary limitations of MIECs lie in their restricted ionic conductivity and the challenges associated with prolonged operation at elevated temperatures. Consequently, composite electrodes typically involve blending the MIEC with electrolyte materials like YSZ and CGO.^78^^,^^79^^,^^86^^,^^87^

Double perovskites, characterized by their complex structures where the B-site cation is substituted by two different types of cations (denoted as B′ and B''), present a unique set of properties that enhance their application in SOECs. Typically formulated as A_2_B'B″X_6_, these materials feature alternating octahedral sites of B'O_6_ and B″O_6_, offering improved flexibility and enhanced physicochemical properties compared to standard ABO_3_ perovskites.^88^ A key example is the Mo-doped SrFeO_3−δ_, which exhibits high sensitivity in its physical properties, such as electrical conductivity, due to the B-site cation order.^89^^,^^90^^,^^91^ Incorporating high-valence Mo ions affects the oxidation state of iron within the perovskite, enhancing oxygen defect formation and influencing both electrical properties and catalytic activity. As Mo concentration increases in the SrFeO_3−δ_ structure under oxidizing conditions, it decreases activation energy for oxygen transport, leading to higher ionic conductivity.^92^^,^^93^ One particular phase of interest is Sr_2_Fe_1.5_Mo_0.5_O_6−δ_, which stands out due to its substantial ionic conductivity of 0.13 S/cm at 800°C in air, significantly surpassing that of many state-of-the-art materials like La_0.6_Sr_0.4_Co_0.2_Fe_0.8_O_3_.^94^^,^^95^ Unlike simpler perovskite materials, Sr_2_Fe_1.5_Mo_0.5_O_6−δ_ benefits from weak Fe–O bonds, which, upon the removal of neutral oxygen atoms, allow for the delocalization of the extra charge across the lattice, thus creating a high concentration of oxygen vacancies.^96^ Despite its impressive properties, Sr_2_Fe_1.5_Mo_0.5_O_6−δ_ does not see widespread use as an oxygen electrode due to synthesis challenges under oxidizing conditions, which limit the solubility of Mo and lead to the formation of an undesirable SrMoO_4_ phase.^97^ Nevertheless, its excellent chemical compatibility with common electrolyte materials (aside from some issues with YSZ) and superior electrochemical performance make it a compelling candidate for specialized applications within SOECs.

The fuel electrode material typically combines ceramic and metallic phases, known as “cermet”. A commonly employed material for fuel electrodes is nickel oxide-yttria-stabilized zirconia (NiO-YSZ). YSZ is renowned for its remarkable stability under reducing conditions and exhibits high ionic conductivity. Upon reduction, metallic nickel assumes a dual function: enabling electronic conduction within the electrode and catalyzing the reduction of hydrogen or reforming hydrocarbon fuels.^98^ Importantly, cermets’ thermal expansion coefficient (TEC) and electrical conductivity vary based on their fabrication processes and resulting microstructure. A specific example includes a Ni-GDC (gadolinium-doped ceria) cermet, which shows a TEC of approximately 14.3 × 10^−6^ K^−1^, demonstrating how properties like TEC can be influenced by variations in Ni content within the composite.^99^ Also, the electrical properties of Ni cermets are significantly affected by factors such as particle size, porosity, and the volume fraction and distribution of Ni and ceramic phases. For example, the conductivity of Ni-YSZ cermets increases with sintering temperature and decreases with increased porosity, showing how manufacturing conditions directly influence functional characteristics.^100^ Moreover, these materials have shown adaptability in different SOC operational modes, effectively handling gas composition variations and operating temperatures that impact their overall performance and stability.^101^

Perovskite-based electrodes, particularly those with MIEC properties, have gained prominence and are explored extensively for their potential as fuel electrodes in H_2_O electrolysis and co-electrolysis settings. The versatility of perovskites is enhanced by doping and mixing different phases, which tailors their electrical and thermal properties to suit specific applications better. For example, LSCM exhibits a simple perovskite structure that can be modified through doping processes. Such modifications significantly influence properties like the TEC, which for LSCM ranges around 9.3 × 10^−6^ K^−1^ across temperatures of 64°C–956°C. Electrical conductivity also varies with the environment; LSCM shows an increase from 7.7 S/cm in the air at 320°C and 1.4 S/cm at 5% H_2_/Ar.^102^

Further, the influence of doping is evident in experiments where Sc was added to LSCM. Sc doping, for instance, decreased the electrical conductivity and significantly reduced the polarization resistance, illustrating the complex interplay between dopant, structure, and performance.^103^ These enhancements facilitate the electrodes' use in demanding operational conditions of SOECs, where factors like temperature, steam content, and gas composition critically impact their efficiency and operational stability. Thus, the strategic manipulation of perovskite-based materials through doping optimizes their physical properties to meet specific operational requirements and emphasizes the potential of tailored material design in advancing SOEC technologies.

## Syngas production and H_2_:CO ratio optimization

### Tuning H_2_:CO ratio via H_2_O:CO_2_ co-electrolysis

Developing cathodes for SOECs that are efficient and stable while also capable of producing syngas with a controllable H_2_:CO ratio is a matter of significant interest. The rationale is obtaining syngas with an adjustable H_2_:CO ratio, enabling its use in downstream sectors.^104^ However, despite several CO_2_ electrochemical conversion strategies being proposed,^105^^,^^106^^,^^107^ relatively little progress has been accomplished with SOEC systems. Thus, the commercial viability of the high-temperature co-electrolysis process depends on controlling the composition of the resulting syngas. In addition, as previously discussed, FT synthesis works with various H_2_:CO ratios, yet other industrial reactions, such as the Oxo synthesis, dimethyl ether, and polycarbonate production, require tunable H_2_:CO ratios.^104^^,^^108^^,^^109^^,^^110^^,^^111^ In this context, it is important to emphasize that conventional natural gas-based plants frequently produce syngas with excess hydrogen, requiring downstream processes such as pressure swing absorption (PSA) units to regulate the H_2_:CO ratios, thereby increasing operating and maintenance costs.^112^^,^^113^ Thus, high-temperature co-electrolysis of H_2_O and CO_2_ presents an opportunity to simultaneously produce syngas and control its composition in a single step.

**Figure 5:**
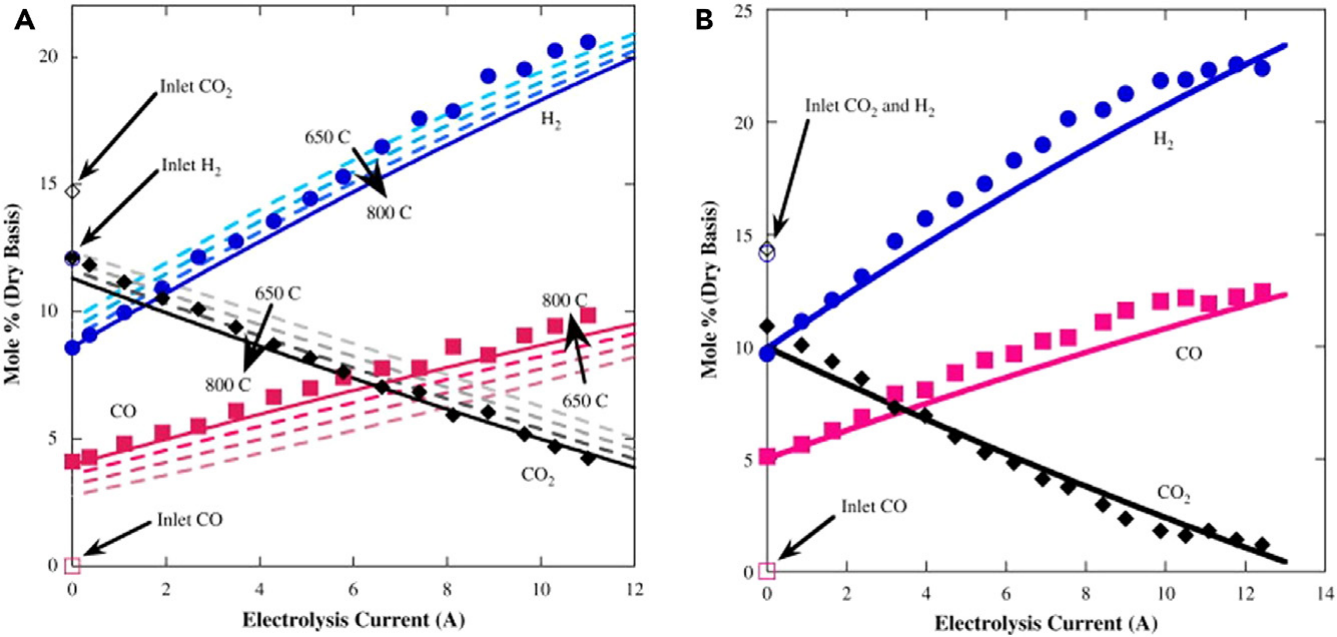
Conditions effect (A) The impact of varying the equilibrium temperature in the chemical equilibrium co-electrolysis model is examined and compared to experimental data. (B) The fraction of outlet gas and CO_2_ conversion rate at 800°C plotted against the electrolysis current obtained from operating the solid oxide co-electrolysis under conditions of 10.2% H_2_, 12.4% CO_2_, 61.9% N_2_, and 15.5% H_2_O. Adapted with permission from ref.^115^; copyright 2009, Elsevier.

Under these circumstances, it is evident that the H_2_:CO ratio of the syngas generated via co-electrolysis can vary depending on factors such as cell voltage, H_2_O:CO_2_ ratio in the cathode feed, temperature, and cathode material properties.^114^ Figure 5 illustrates the impact of temperature on the composition of the outlet gas. It is evident that raising the operating temperature enhances CO production but detrimentally affects H_2_ production. This contrasting temperature effect on CO and H_2_ production likely arises from the H_2_ generation through steam splitting and CO production primarily via the RWGS reaction. The RWGS reaction, being temperature-dependent, favors CO production by consuming H_2_. Consequently, the chemical equilibrium model can forecast syngas composition by adjusting key operating parameters, including inlet gas composition, operating temperature, and electrolysis current.^115^

**Figure 6:**
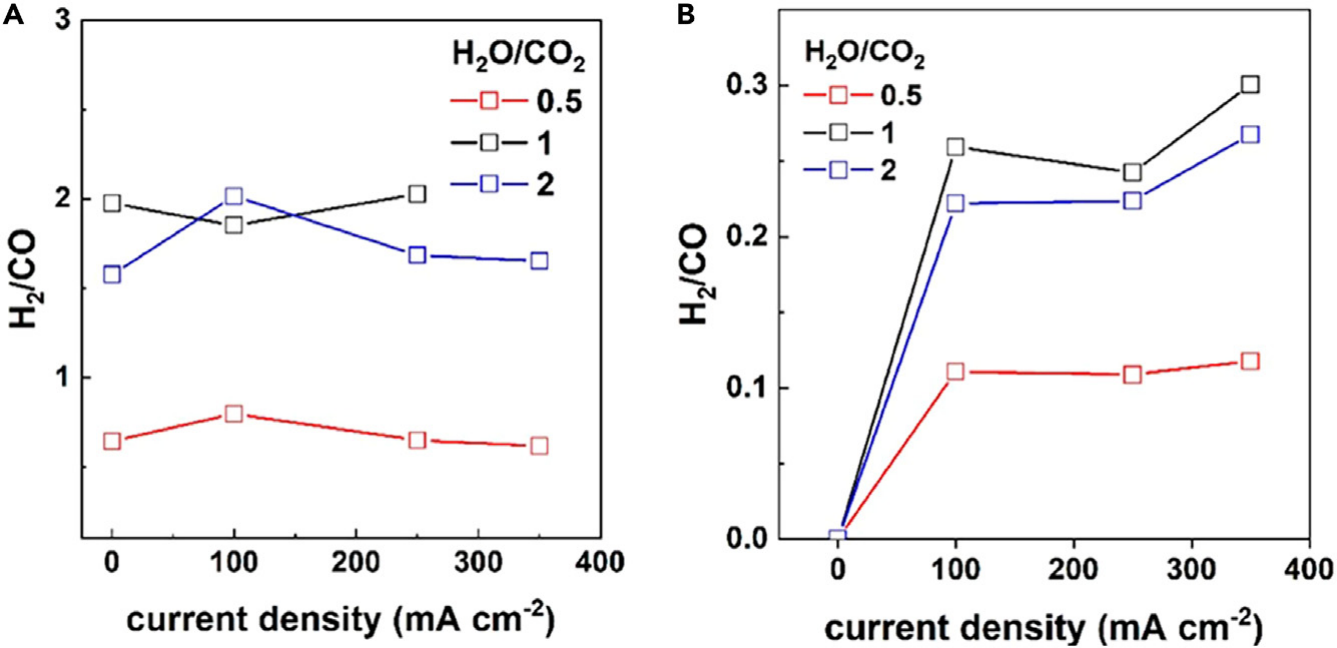
Steam/carbon dioxide ratio effect Ratio of H_2_:CO, as calculated from the outlet analysis, under open circuit and electrolysis conditions for three H_2_O:CO_2_ ratios: 0.5, 1, and 2 when H_2_ is (A) present and (B) absent from the feed, at 900°C. Reproduced with permission from ref.^116^; copyright 2023, MDPI.

**Figure 7:**
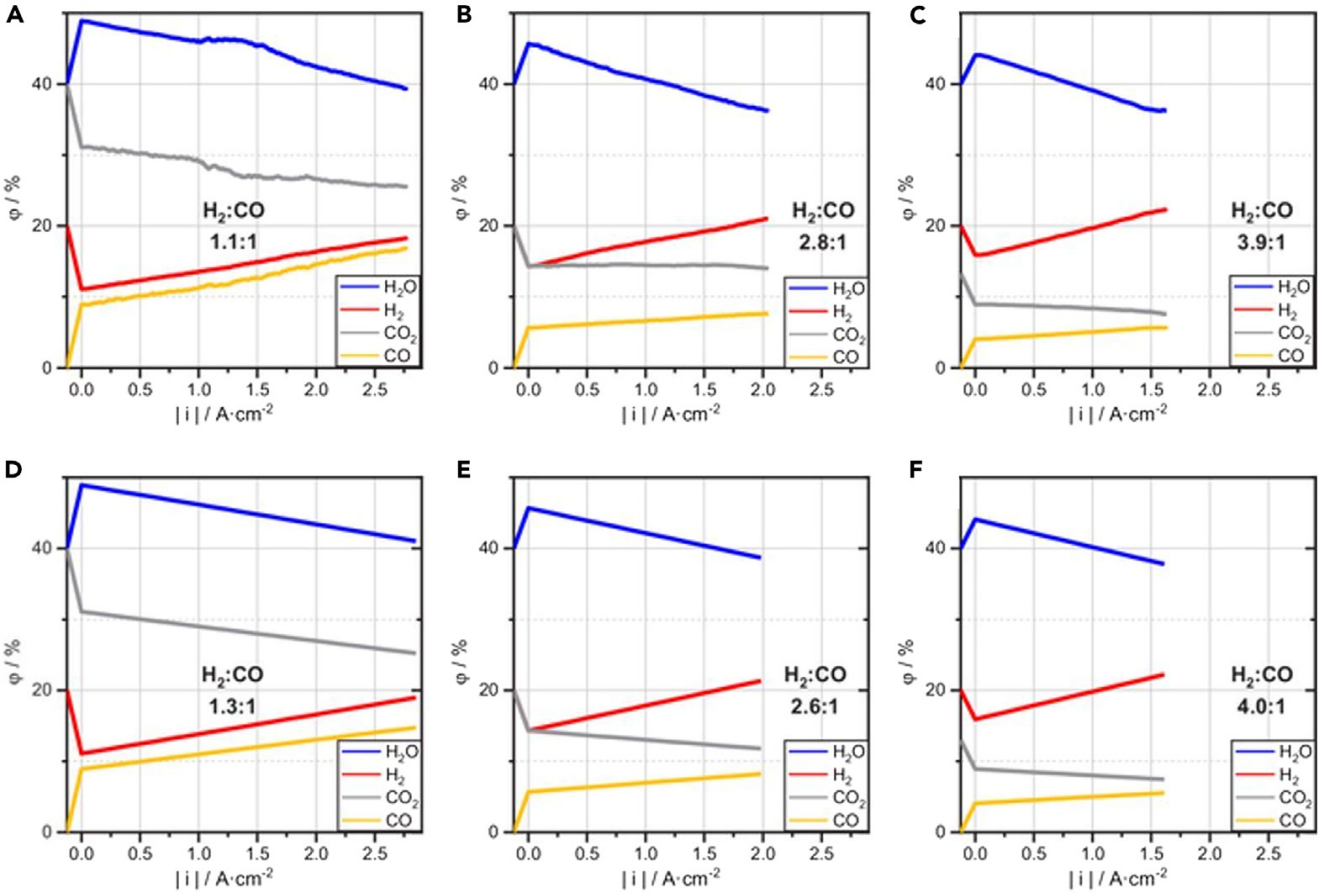
Comparison between experimental and theoretical product compositions (A–C) Experimental and (D–F) theoretical product gas compositions are depicted as a function of current density. The initial compositions of 40% H_2_O + 40% CO_2_ + 20% H_2_ (A and D), 40% H_2_O + 20% CO_2_ + 20% H_2_ + 20% N_2_ (B and E), and 40% H_2_O + 13% CO_2_ + 20% H_2_ + 27% N_2_ (C and F) at 900°C and 6 L h^−1^ are compared. The experiments were conducted up to a potential of 1.4 V, resulting in differing current density limits. Reproduced with permission from ref.^117^; copyright 2019, IOP Science.

Bimpiri et al. investigated how varying the steam/carbon dioxide ratio (H_2_O:CO_2_ ratio = 0.5, 1, and 2) in the feed affects the electrochemical performance at 900°C. Using an 8YSZ electrolyte-supported cell with iron-doped lanthanum strontium chromite as the fuel electrode material and lanthanum strontium manganite as the oxygen electrode, it was observed that the H_2_:CO ratio was higher when H_2_O:CO_2_ equals 1 and 2 compared to a lower H_2_O:CO_2_ ratio of 0.5 (Figure 6). They highlighted that these results persisted regardless of whether H_2_ was simultaneously fed to the cathode by comparing Figures 7A and 7B. Yet, elevating the current density from 100 to 350 mA cm^−2^ exhibited only a minimal impact on the H_2_:CO ratio.^116^ From an economic standpoint, the reduced need for such a high current density means lower energy consumption for the same output, enhancing the financial viability of syngas production and contributing to the overall sustainability of the process, making it more attractive for industrial-scale applications.

Dittrich et al. also explored high-temperature co-electrolysis of CO_2_ and steam as a promising system for producing renewable energy syngas. The components of the cells include a cathode made from nickel and yttria-stabilized zirconia (Ni/YSZ), a YSZ electrolyte, a cerium gadolinium oxide (CGO) barrier layer, and a lanthanum strontium cobaltite-ferrite (LSCF) anode. Such a configuration ensures efficient electrolysis by optimizing conductivity and chemical stability. They demonstrated the existence of a linear relationship between the H_2_O:CO_2_ ratio in the feed gas and the H_2_:CO ratio in the output gas, i.e., by adjusting the H_2_O:CO_2_ ratio in the feed gas, they could precisely tailor the syngas ratios in a single electrolysis step. Their approach yielded syngas compositions that closely matched the input feed gas ratios, with a consistently slight excess of hydrogen observed across the tested compositions. Moreover, their findings highlight the flexibility and control offered by this method of syngas production.^117^

Moreover, the study showed that the syngas ratio remains mostly consistent despite variations in the electrochemical potential and gas utilization rates during operation. Additionally, they showed that the composition of syngas remains consistent regardless of changes in current density. This stability ensures that co-electrolysis processes are well-suited to operate efficiently under the variable conditions renewable energies provide.^117^ In Figure 7, various feed gas compositions are employed, and the outlet gas composition is displayed on the y axis. The current is measured incrementally until a voltage of 1.4 V is reached. Figure 7A exhibits the highest CO_2_ content and a 1:1 ratio of H_2_O:CO_2_. Due to its high fuel content, it demonstrates a higher current density compared to Figures 7B and 7C, which use less CO_2_ and achieve lower current densities. The theoretically calculated compositions in Figures 7D–7F closely align with the experimental data.

Studies have also investigated the impact of cathode composition and design on the resulting H_2_:CO ratio. For instance, Deka and co-workers evaluated the cathode composition and architectural influence of Ni and Co-doped perovskite materials as SOEC cathodes for co-electrolysis of CO_2_ and H_2_O at 800°C. The study explored surface and bulk properties modifications due to doping and demonstrated that the H_2_:CO ratio in the produced syngas increases with increasing Ni content in the cathode material. This ratio could be controlled by adjusting B-site dopant levels, cell voltage, and the H_2_O:CO_2_ ratio in the cathode feed stream. The study also identified that Co-doped cathodes might form graphitic carbon, reducing Faradaic efficiency for syngas production, while Ni-doped cathodes do not exhibit such an issue. Long-term co-electrolysis tests show good stability of the materials in terms of electrochemical performance and coke resistance.^114^

**Figure 8:**
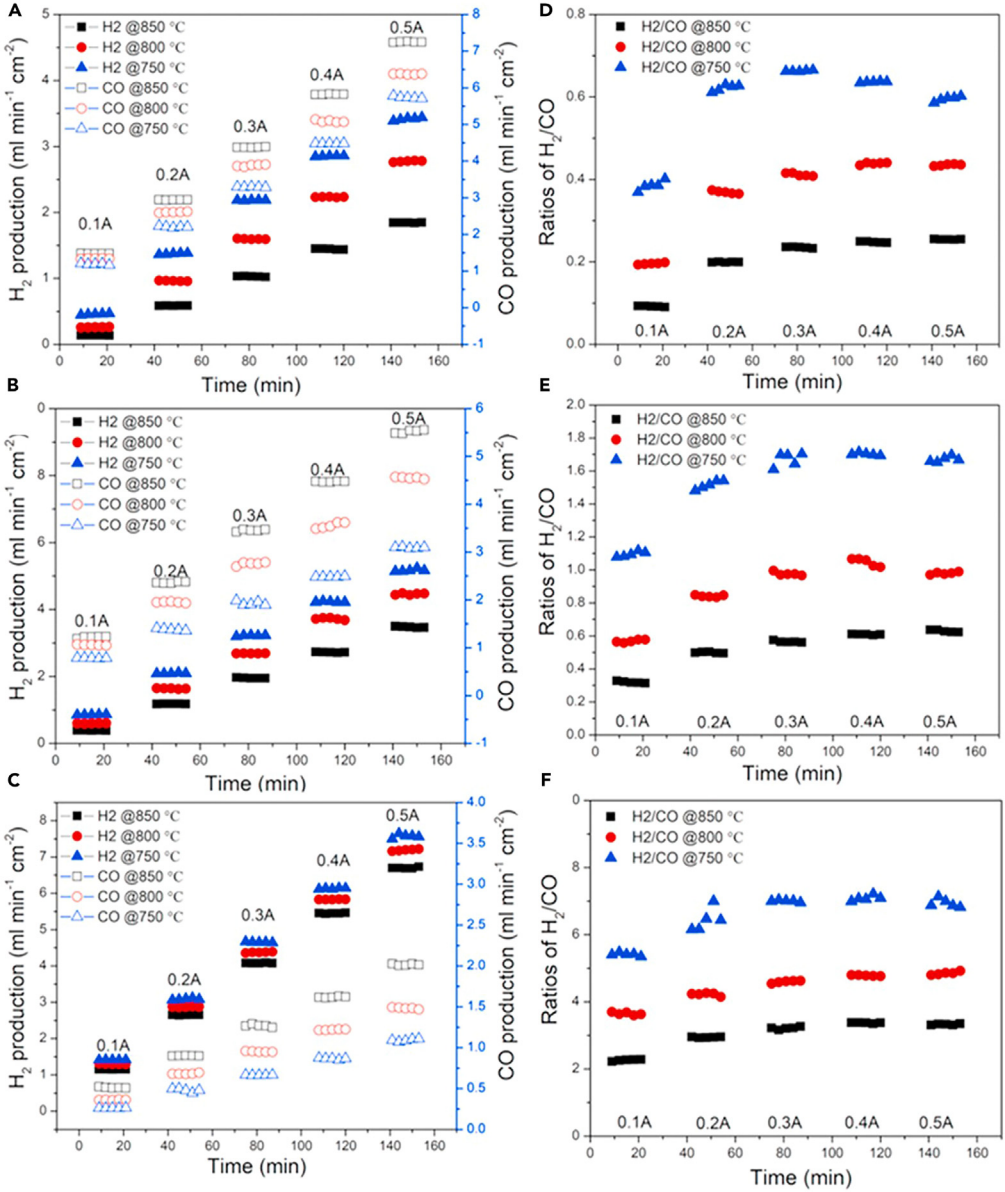
Manipulating operation conditions Experimental data from online gas chromatography display (A–C) H_2_ and CO_2_ production and (D–F) the ratio of H_2_:CO under various ratios of H_2_O:CO_2_ and different conditions. (A and D) represent 10% H_2_O + 90% CO_2_; (B) and (E) represent 20% H_2_O + 80% CO_2_; and (C and F) represent 50% H_2_O + 50% CO_2_. Reproduced with permission from ref.^118^; copyright 2021, Elsevier.

Bian et al. achieved high-rate production of syngas with a tunable H_2_:CO ratio and coke-free operation in an SOEC through controlled pre-reduction of the La_0.7_Sr_0.3_Fe_0.9_Ni_0.1_O_3-δ_ (LSFNi) cathode, in which the *in-situ* exsolution of Ni-Fe alloy nanoparticles facilitated efficient co-electrolysis of H_2_O and CO_2_ to H_2_ and CO. Current densities of up to 1.0 A cm^−2^ at 750°C and 2.4 A cm^−2^ at 850°C were attained at 1.5 V, with near 100% Faradaic Efficiency. As exhibited in Figure 8, by manipulating the H_2_O:CO_2_ ratio of the feed gas, operating temperature, and current density, they demonstrated the feasibility of adjusting the output H_2_:CO ratio by nearly two orders of magnitude, ranging from approximately 0.1 to about 7. Moreover, it was demonstrated that with a 1:1 volume ratio of H_2_O:CO_2_ at 850°C and under a current as low as 0.1 A, a ratio of 2 was achieved, which is close to the optimal ratio required for Fischer-Tropsch reactions. Stable operation for over 100 h at 800°C was attained without any evidence of carbon deposition, although operating at high current density led to observable deterioration of the anode/electrolyte interface due to rapid oxygen evolution.^118^

Maide et al. also focused on a redox exsolution growth of a nickel catalyst on the surface of a La_1-x_Sr_x_Cr_1-y_Mn_y_O_3-δ_ (LSCM) electrode with 6% Ni on the B-site, impregnated onto a porous matrix composed of Sc0_.1_Ce_0.01_Zr_0.89_O_2-δ_. The porosity of the matrices was evaluated, and ICP-MS analysis was conducted to determine the correct cation stoichiometry of the raw solutions. The research found that deficiency in the A-site led to an increase in the ohmic component of resistance and a decrease in charge transfer resistance. It was also observed that charge transfer resistance decreased sequentially from pure LSCM to LSCMN, with greater A-site deficiency as the Ni catalyst concentration on the surface increased. The study suggests that further improvements in the performance of LSCMN electrodes could be achieved through additional optimization of electrode microstructure and stoichiometry.

**Figure 9:**
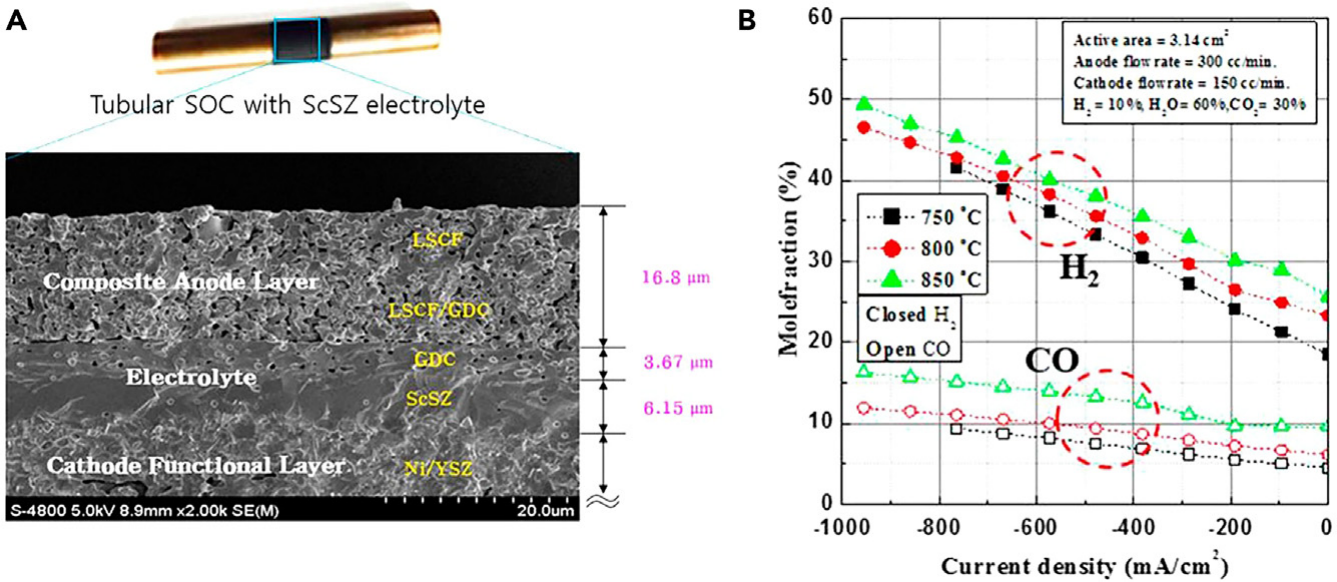
SEM evaluation and analysis of the syngas composition (A) SEM image depicts the cross-section microstructure of tubular co-electrolysis cells based on ScSZ electrolyte and (B) analysis of syngas composition after co-electrolysis on a tubular SOC at an operating temperature of 750°C–850°C. Adapted with permission from ref.^119^; copyright 2018, Elsevier.

Yu et al. used tubular SOECs consisting of fuel-electrode-supported cells based on Ni-yttria stabilized zirconia (Ni-YSZ), an electrolyte made of scandia-stabilized zirconia (ScSZ), and a composite air-electrode composed of a lanthanum strontium cobaltite-ferrite (LSCF). The ScSZ electrolyte and GDC interlayer were applied onto the fuel-electrode-support electrode surface using vacuum-slurry and dip-coating techniques. Sequentially, GDC-LSCF and LSCF were also applied as composite air-electrode layers (Figure 9A). They investigated the impact of varying operating temperatures on syngas production through co-electrolysis. They maintained a constant inlet gas composition (H_2_O 60%, CO_2_ 30%, H_2_ 10%) while adjusting the temperatures from 750°C to 850°C. Results showed that increasing current density led to a more significant increase in H_2_ mole fraction than CO (Figure 9B). This disparity suggests that H_2_O electrolysis is more efficient than CO_2_ electrolysis as it requires lower voltage. At 850°C, the CO mole fraction increased remarkably, indicating the influence of the water-gas shift reaction (RWGS). This observation suggests that RWGS dominates CO production rather than electrochemical reactions.^119^

Based on the above, it is evident that there is still much work to develop strategies for SOECs to achieve high efficiency, stability, and controllable syngas production with adjustable H_2_:CO ratios. While there is significant interest in obtaining syngas with tunable ratios within a single reactor, current strategies have not yet led to substantial advancements in SOEC systems. The commercial viability of high-temperature co-electrolysis processes largely depends on achieving desired syngas compositions. Although various industrial reactions require specific H_2_:CO ratios, existing natural gas-based plants often produce syngas with excess hydrogen, necessitating additional downstream processes and increasing operational costs. Thus, the potential of high-temperature co-electrolysis lies in its ability to simultaneously produce syngas and control its composition in a single step. However, achieving this goal requires further exploration and optimization of factors such as cell voltage, operating conditions, cathode material properties, and feed gas compositions. Research efforts have demonstrated promising approaches, but challenges such as electrode stability, Faradaic efficiency, and long-term performance still need to be addressed to make high-temperature co-electrolysis a viable and sustainable option for industrial-scale syngas production.

### CH_4_-assisted steam electrolysis in SOEC

The research conducted by Joel Martinez-Frias et al. introduced a pioneering approach to H_2_ production through a solid oxide natural gas-assisted steam electrolyzer.^120^ The system reduced electricity consumption by utilizing natural gas to react with the produced oxygen during steam electrolysis, lowering the chemical potential and enhancing efficiency. A notable breakthrough in their study was achieving up to 1 V reduction in single-cell experiments compared to conventional steam electrolyzers. Furthermore, integrating the electrolyzer with a heat recovery system, comprising heat exchangers and a catalytic reactor achieved system efficiencies of up to 70% relative to primary energy. Consequently, ongoing research is focused on further exploring natural gas-assisted electrolysis systems, aiming to enhance the competitiveness of SOECs compared to CH_4_ steam reforming (MSR) for H_2_ production (Reaction (5)):

(Equation 5) $${\text{CH}}_{4}\;\left(g\right)+H_{2}O\;\left(g\right)\;⇌\;3H_{2}+\text{CO}\;\left(g\right)$$

Interestingly, these systems operate in a hybrid mode, utilizing electricity and fuel oxidation, effectively lowering the thermodynamic potential by exposing the anode to natural gas or CH_4_, which can directly react with oxygen ions to produce CO and H_2_.^121^ Notably, this approach enhances the efficiency of H_2_ production and offers environmental benefits.^122^^,^^123^ Additionally, by integrating renewable energy sources into the electricity supply for electrolysis, these systems have the potential to further reduce carbon emissions and accelerate the transition toward a greener energy landscape.

The development of such integrated systems represents significant progress in H_2_ production. However, the primary focus of this review is on syngas production via SOECs for integration with FT processes. Thus, the development of methane-assisted SOECs for H_2_O:CO_2_ co-electrolysis systems holds immense promise for enhancing syngas formation while simultaneously reducing energy input requirements. Consequently, these systems will be further explored in this article.

#### High-temperature H_2_O:CO_2_ co-electrolysis integration with POM

When dealing with syngas production, MSR finds widespread application in SOFC systems, particularly in direct internal reforming. In this setup, the heat generated by the fuel cell’s exothermic reaction and the steam produced are directly utilized for methane reforming.^124^^,^^125^ A complete MSR typically yields syngas with an H_2_:CO ratio of approximately 3.^126^^,^^127^ Methane dry reforming (MDR) generates a syngas mixture with an approximate H_2_:CO ratio of 1, according to Reaction (6).

(Equation 6) $${\text{CH}}_{4}\;\left(g\right)+{\text{CO}}_{2}\;\left(g\right)\;⇌\;2H_{2}+2\text{CO}\;\left(g\right)$$

Furthermore, RWGS reactions frequently impact the resultant composition, where a portion of the hydrogen is utilized in water production. Consequently, the ultimate H_2_:CO ratio resulting from MDR tends to be lower than one.^128^^,^^129^^,^^130^ An appealing alternative would be to produce syngas H_2_:CO through partial oxidation using oxygen as the oxidant. As far as we can tell, the “Natural Gas-Assisted Steam Electrolyzer” patent granted to Ai-Quoc Pham, Henrik Wallman, and Robert P. S. Glass represents for the first time the H_2_ production (U.S. Patent Number 6051125 on April 18, 2000) by high-temperature steam electrolysis integrating natural gas assistance, substantially decreasing the electrical energy required.^131^ The novel aspect of the patent was the utilization of a catalyst (which can be composed of materials selected from the group consisting of nickel (Ni) cermets, rhodium (Rh), and ruthenium (Ru)) on the anode side of the electrolyzer, which promotes the partial oxidation of natural gas to CO and H_2_. The patent details two main examples of the natural gas-assisted steam electrolyzer. The first involves partially oxidizing natural gas to produce syngas and hydrogen, significantly reducing the system’s electricity consumption. The second approach utilizes natural gas to burn out the oxygen produced during electrolysis, minimizing or eliminating the potential difference across the electrolyzer membrane and reducing electricity use further.

POM reaction, shown in Reaction (7), exhibits mild exothermicity, providing a means to offset the energy requirements of the co-electrolysis process.^56^

(Equation 7) $${\text{CH}}_{4}+½O_{2}=\text{CO}+2H_{2}$$

In contrast to MSR and MDR, POM offers the distinctive advantage of producing an H_2_:CO mixture with an ideal ratio of approximately 2:1 and has, therefore, been considered for the direct production of syngas via electrochemically assisted processes using SOEC devices.^132^ Thus, integrating POM with solid oxide electrolysis facilitates electrical-to-chemical energy conversion, offering a sustainable energy storage and load-balancing solution. The rationale behind these innovative studies is that an integrated SOEC/POM device holds significant potential advantages due to the moderately exothermic characteristic of the POM reaction (Δ_Ho_ = −802 kJ mol^−1^), which can offset the energetic demands of co-electrolysis.^133^

In addition, such integration offers a synergistic approach for efficient syngas production once this combination utilizes the high-temperature capability of SOECs to enhance the POM reaction, converting methane and a limited amount of oxygen into syngas. The advantages of this integration include improved energy efficiency, reduction of CO_2_ emissions, and the production of a valuable precursor for synthetic fuels and chemicals, all contributing to a more sustainable energy landscape.

It is important to note that numerous reactions compete during syngas production via POM. The preferred pathway depends on various operating parameters (e.g., temperature, catalyst type). Establishing the correct thermodynamic window is crucial for the system’s proper operation. Side reactions such as the Boudouard reaction (Reaction (8)) and methane decomposition (Reaction (9)) can be highly detrimental to the electrode due to carbon deposition.^134^

(Equation 8) $$2\text{CO}\;\to \;C_{\left(s\right)}+{\text{CO}}_{2}$$

(Equation 9) $${\text{CH}}_{4}\;\to \;C_{\left(s\right)}+2H_{2}$$

In the conventional mode of solid oxide electrolysis, a significant portion of the electrical power is utilized to overcome the high chemical potential required to facilitate oxygen diffusion through the electrolyte. An interesting paper from Wang and Hang introduced a novel approach where a methane-assisted H_2_O:CO_2_ co-electrolyzer was devised by integrating an SOEC with POM to produce syngas on both sides. In the POM-SOEC mode, CH_4_ was introduced to the oxygen anode to react with the oxygen generated during the electrolysis process, thereby reducing the chemical potential. Experimental results conducted on the NiYSZ/YSZ/SFM-YSZ single cell demonstrated a significant reduction in voltage from 1.3 V to 0.3 V compared to the conventional electrolysis mode at the same current density.^135^

Cui et al. introduced symmetric YSZ-LSCrF | YSZ | YSZ-LSCrF cells, enhanced with Ni-SDC catalysts, designed for methane-enhanced co-electrolysis of H_2_O and CO_2_. In the study, to achieve an electrolysis current density of −400 mA cm^−2^ at a temperature of 850°C, the electrical voltage required is significantly lowered from 1.0 V in the case of traditional co-electrolysis to just 0.3 V when employing methane-enhanced co-electrolysis. This reduction signifies a remarkable 70% cut in the energy consumption for the process. This advancement showcases the efficiency of incorporating methane into the co-electrolysis process and represents a leap forward in making syngas more sustainable and cost-effective.

**Figure 10:**
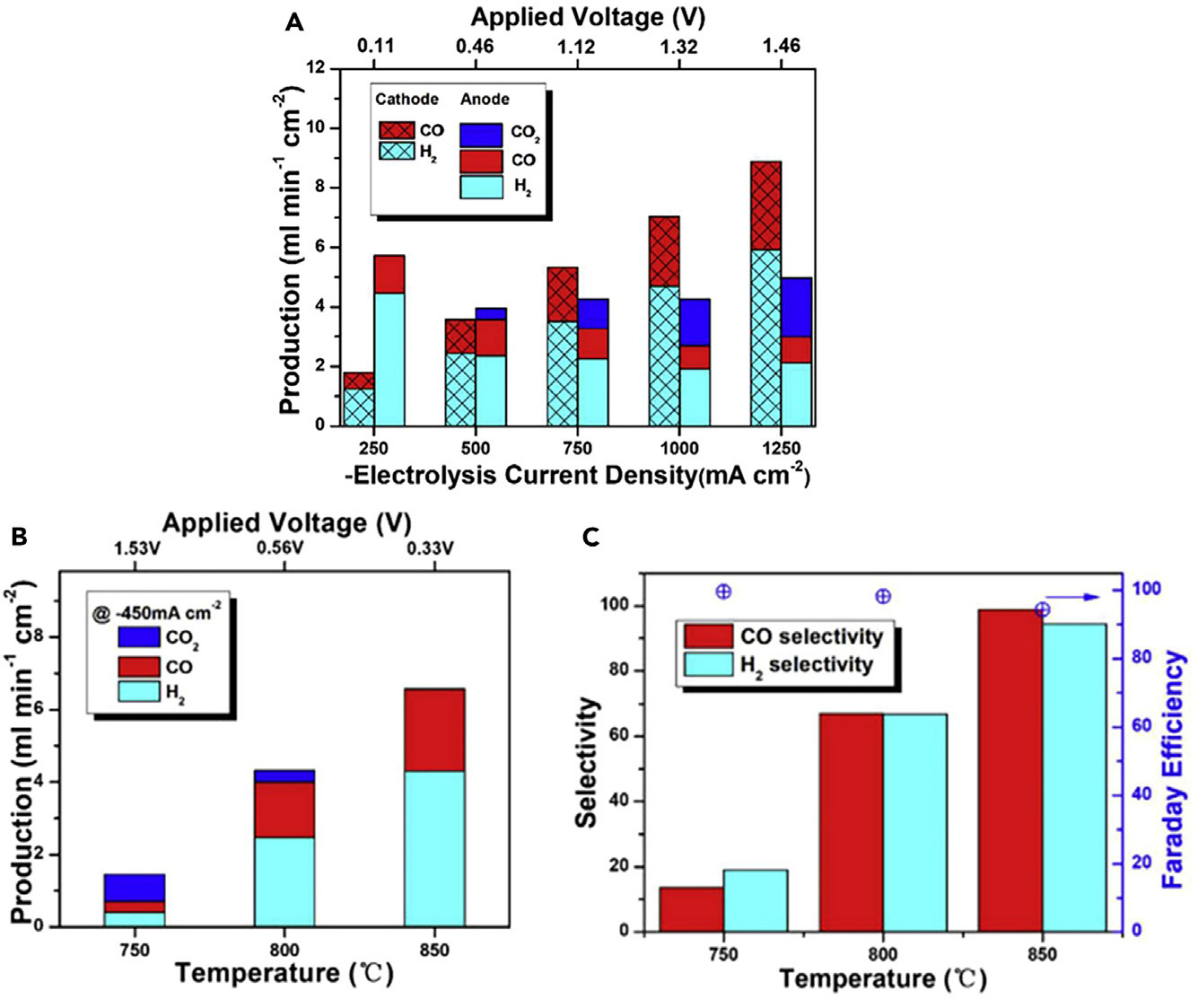
Outlet gas composition (A) Outlet gas composition from both electrodes measured under different electrolysis current densities at 850°C, (B) Outlet gas composition at the anode at different operating temperatures of 750°C–850°C and current density of −450 mA cm^−2^, and (C) CO and H_2_ selectivity of the outlet gas composition of the anode and Faraday efficiency at different operating temperatures of 750°C–850°C and current density of - 450 mA cm^−2^. Adapted with permission from ref.^136^; copyright 2021, Science Direct.

In the study on the cathode, a 1:1 volume ratio of H_2_O and CO_2_ input consistently yielded syngas with an H_2_:CO ratio of approximately 2 across various current densities. However, the composition of the gas exiting the anode, where CH_4_ is introduced, varies with the current density, showing a strong dependence of methane oxidation products on the applied electrolysis current densities. In fact, operations above critical current densities were required to avoid coking formation. On the other hand, the CO_2_ formation rate started to increase pronouncedly with the electrolysis current densities above −500 mA cm^−2^ (Figure 10A). Strong temperature dependence is also observed, as depicted in Figures 10B and 10C, where high temperature exhibits better selectivity but lower Faradaic efficiency. The study indicated a preference for syngas production under moderate current densities at elevated temperatures. Specifically, at 850°C and an electrolysis current density of −450 mA cm^−2^, the anode can generate syngas with an H_2_:CO ratio of about 2 at a rate of 6.5 mL min^−1^.cm^−2^.^136^

The SOEC can also operate with just CO_2_ electrolysis and POM, although it is less common. Sun et al. discuss such an approach, which enables the direct production of syngas at the anode side. The researchers describe the SOEC configuration, where CO_2_ (to produce CO) is injected into the cathode chamber, and diluted CH_4_ is injected into the anode chamber to initiate POM with the oxygen species generated from CO_2_ electrolysis. Experimental results demonstrate the effectiveness of this process across various temperatures and conditions, highlighting the capability to adjust syngas ratios by controlling the outlet flow rates. Additionally, the study addresses the challenges of CH_4_ conversion and CO selectivity, emphasizing the importance of optimizing electrode catalytic activities and the SOEC configuration for improved efficiency.^137^

## FT synthesis for liquid fuel production

The FT synthesis, initially elucidated in 1923 by Franz Fischer, Hans Tropsch, and Helmut Pichler at the Kaiser Wilhelm Institute, is a remarkable catalytic process for synthesizing hydrocarbons from syngas. The pioneering research involved a cobalt-based catalyst, resulting in the production of gasoline, diesel, and medium to high molecular weight distilled oils.^138^ The first industrial-scale FT reactor was implemented by Ruhrchemie in 1935, configured as an atmospheric fixed bed reactor, achieving an annual capacity of 100,000–120,000 metric tons. Currently, the reactors in operation are of the conventional fluidized bed type, designated as Sasol Advanced Synthol (SAS) reactors. The largest among them boasts a capacity of 20,000 barrels per day. The process predominantly produces gasoline, diesel, LPG, and some oxygenated hydrocarbon products.^139^ Thus, associating the ability of SOECs to generate syngas and the proven capability of the FT process to convert syngas into a wide range of hydrocarbon products represents a promising path for producing sustainable and carbon-neutral hydrocarbons. Although FT synthesis typically does not aim to produce CH_4_ as a primary product, the gas is inevitably produced. The formation of CH_4_ in the FT process is generally considered a side reaction, occurring via the hydrogenation of CO or as a result of the methanation of CO and CO_2_ present in the syngas (Reaction (10)).

(Equation 10) $$\text{CO}+3H_{2}\;\to \;{\text{CH}}_{4}+H_{2}O$$

Shell’s incursion into Bintulu, Malaysia, in 1993 saw the establishment of an FT plant based on CH_4_, which utilizes syngas generated by non-catalytic POM at pressures up to 70 bar and temperatures close to 1400°C. The four large-scale multitubular reactors employ a cobalt-based catalyst, each with a capacity of approximately 125,000 tons per year.^140^ However, integrating FT synthesis with SOEC allows the production of CH_4_ to be part of the fuel-assisted electrolysis.^141^ In addition, the formulation of catalysts to work with syngas variation is important to optimize the efficiency and selectivity of catalytic processes. The presence of different components, such as CO_2_ and H_2_O, can affect the catalytic activity, stability, and lifetime of the catalyst. Although the FT reaction is catalyzed by metals such as iron and cobalt, occurring at pressure ranges between 10 and 60 bar, with temperatures varying between 200°C and 300°C,^142^ the current literature does not provide much information on the optimized formulation of catalysts to specifically deal with the variation in syngas composition provided by SOECs. Iron-based catalysts could be a promising option for high-temperature FT synthesis.

### Development of CH_4_-assisted SOEC for H_2_O:CO_2_ co-electrolysis systems for FT integration

**Figure 11:**
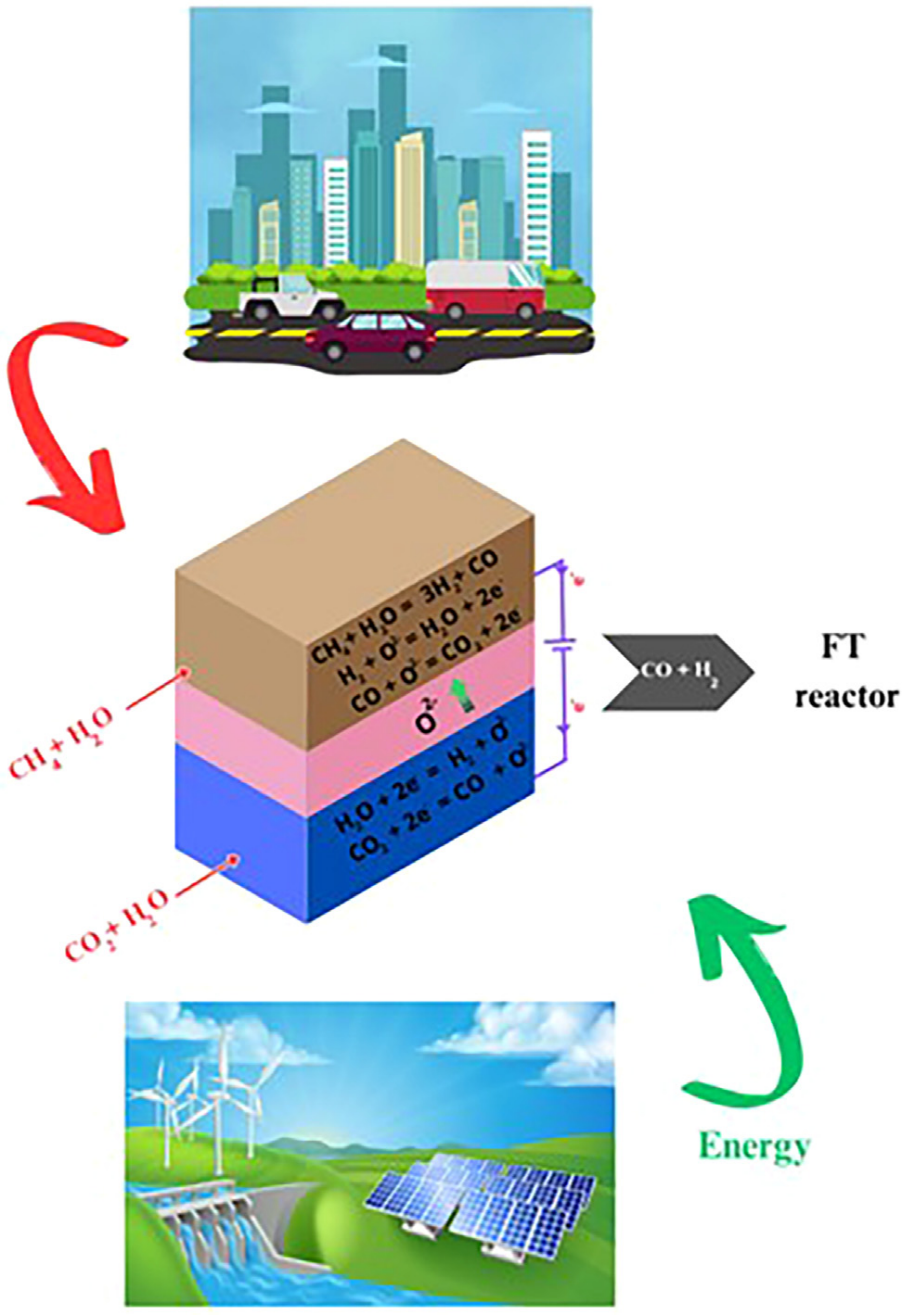
Illustration of a system integrating a CH_4_-assisted SOEC with an FT reactor

Designing CH_4_-assisted SOEC for H_2_O:CO_2_ co-electrolysis systems represents a pioneering advancement toward sustainable fuel production. Such an innovative system promises to combine SOEC technology and FT synthesis, combining CH_4_ utilization as an efficient fuel to enhance the SOECs' performance while minimizing electricity consumption.^143^ Such a strategic approach reduces operational costs and significantly boosts the system’s efficiency compared to traditional SOECs.^51^ Integrating CH_4_-assisted SOEC and FT synthesis presents a formidable solution for low-carbon fuel generation, promising a significant reduction in CO_2_ emissions (Figure 11). Basically, CH_4_ or CH_4_ and H_2_O are introduced to the anode; concurrently, CO_2_ and H_2_O are fed into the cathode side for electrolysis, producing syngas. This produced syngas is then directed to the FT reactor, which undergoes a synthesis process to form hydrocarbons.

Although very interesting, only a few studies are available in this area, and they are centered on the simulation aspects of the process to understand and optimize the process parameters that affect the system’s performance. Such an approach has laid a solid foundation for understanding the complexities of CH_4_-assisted SOEC for H_2_O:CO_2_ co-electrolysis and FT synthesis systems. The so-called Power-to-Liquid pathway converts CO_2_ into synthetic value-added products by integrating carbon capture and utilization strategy. Simultaneously, this approach explores the production of fuels and chemicals through the FT process. However, it identifies biogas upgrading, RWGS, and SOEC synergy with FT synthesis as the most promising candidates. Nevertheless, Marchese et al. unveiled that specific configuration could attain up to 81.1% plant efficiencies with SOEC as the syngas generator and 71.8% for the RWGS option, alongside global carbon reduction potentials of 79.4% and 81.7%, respectively. Such insights emphasize the significant potential of integrating SOEC and FT technologies. By presenting a detailed process modeling that includes energy integration and optimization, the study offers profound insights into the design and operational dynamics of such integrated systems, promoting a more sustainable approach to chemical production.^144^

Another simulation study investigated the potential of enhancing SOECs through CH_4_ assistance, showing a significant lowering of the equilibrium potential of the cell, thereby reducing the overall electrical energy requirement for the co-electrolysis of H_2_O and CO_2_. The method was numerically analyzed using a 2D model, validated against experimental H_2_O:CO_2_ co-electrolysis data. Furthermore, the study explored the impact of CH_4_ concentration and gas flow rates on performance; their findings indicate that optimizing these parameters can further improve the system’s efficiency, highlighting the potential for design and operational enhancements in SOECs employing CH_4_ assistance.^51^

Recent studies have explored this innovative approach, emphasizing its potential in carbon recycling and synthetic fuel generation. Modeling studies present the viability and efficiency of integrating CH_4_-assisted SOECs with FT synthesis. The study highlights the significance of optimizing the syngas composition, achieved through the co-electrolysis of CO_2_ and H_2_O. They introduced a 2D model to assess the performance and output of this integrated system, focusing on the parametric effects on syngas production and subsequent hydrocarbon synthesis. They demonstrated how adjusting the H_2_O:CO_2_ ratio at the SOEC’s cathode inlet can significantly influence the composition of produced syngas, thereby affecting the efficiency and economic feasibility of generating value-added hydrocarbon fuels.^141^

Similarly, they showed how CH_4_ reforming and electrochemical reactions within SOECs can be exploited to reduce electricity consumption while enhancing syngas production. They further validated the system’s modeling through detailed parametric analyses and explored the operational parameters that impact fuel synthesis. Thus, the study emphasized the importance of controlling the H_2_:CO ratio in syngas for optimizing the FT synthesis process, aligning with the findings of the first article regarding syngas composition management.^145^ Connecting the findings of both studies, it becomes evident that the integrated approach not only promises a reduction in CO_2_ emissions through its utilization but also offers a pathway to economically viable and sustainable fuel production. The research demonstrates that syngas production efficiency can be significantly enhanced through careful adjustment of operational parameters, such as the inlet H_2_O:CO_2_ ratio and the integration of CH_4_ assistance. This optimization is crucial for the downstream FT synthesis process, where syngas' quality directly influences the produced hydrocarbons' yield and quality.

Thus, the integrated CH_4_-assisted SOEC and FT synthesis offers a promising path for the sustainable production of synthetic fuels, emphasizing the importance of syngas composition optimization and operational parameter adjustments. This approach promises a reduction in CO_2_ emissions and opens the door for economically viable and sustainable fuel production. In addition, integrating SOEC with FT synthesis processes offers promising pathways for improving synthetic fuel production’s overall efficiency and sustainability. For example, Kuznetsov et al. explored the integration of a high-temperature co-electrolysis unit with the FT synthesis process. This approach controls by-products from the FT process as an energy source for the co-electrolysis unit, producing syngas from CO_2_. By adjusting process stream temperatures and optimizing utility systems, this integration significantly reduces energy consumption and carbon emissions associated with syngas production, aligning economic benefits with environmental sustainability.^146^ Hence, this integration can reduce energy inputs, enhance process efficiencies, and minimize environmental impact, demonstrating a cohesive approach to more sustainable and efficient synthetic fuel production technologies.

## Challenges, degradation mechanisms, and mitigation strategies

Although SOECs offer high efficiency, the degradation they experience establishes significant challenges that hinder their widespread adoption and industrial use.^147^ Degradation processes affect SOECs by reducing their performance over time, thereby increasing energy consumption for the same output, which raises operational costs. Indeed, no deep studies regarding methane-assisted processes are available in the literature; thus, we decided to overview SOEC issues, which could help other processes.

Hence, there is a need for frequent maintenance and component replacement due to issues like electrode delamination, phase changes at interfaces, and electrolyte deterioration.^147^^,^^148^^,^^149^^,^^150^ Moreover, given their susceptibility to rapid degradation under typical operating conditions, SOECs have shorter operational lifespans than other technologies that can sustain longer service periods without significant performance loss, as discussed in the introduction section. Therefore, enhancing the commercial viability of SOECs involves developing materials that resist degradation, improving cell designs to mitigate its effects, and developing maintenance strategies that minimize operational disruptions and costs.

**Figure 12:**
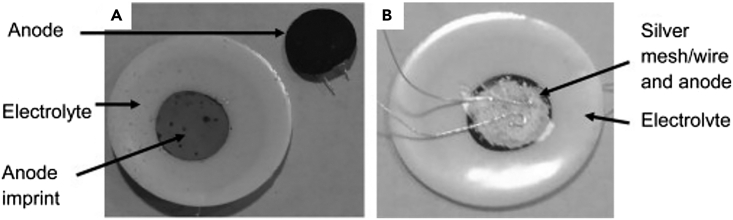
Degradation phenomena (A) Following a 100-h test at 0.8 V, the LSM anode was found to have separated from the YSZ electrolyte. (B) A symmetric cell in its untested state featuring an electrolyte disk with screen-printed electrodes. The cell assembly also includes a silver mesh and wires. Adapted with permission from^152^; copyright 2012, Science Direct.

The degradation phenomena and mechanisms of SOECs are complex and significantly impact the efficiency and longevity of these systems.^151^ Degradation at the oxygen electrode, particularly for materials such as LSM and LSCF, is one of the most critical challenges.^152^^,^^153^^,^^154^^,^^155^ For LSM, a commonly observed degradation mechanism is the delamination from the yttrium-stabilized zirconia (YSZ) electrolyte, aggravated by the formation of detrimental phases such as La_2_Zr_2_O_7_ under high operating currents. This phase formation results from reactions between the LSM and the ZrO_2_ in the electrolyte, driven by high local stresses and strains at the electrode/electrolyte interface^156^ For example, one can notice the delamination process of an LSM anode YSZ electrolyte after 100 h testing at 0.8 V (Figure 12A), leaving a dark coloration. In contrast, the cathode layers from the cells not subjected to testing demonstrated robust adherence to the electrolyte, as evidenced in Figure 12B.^152^

**Figure 13:**
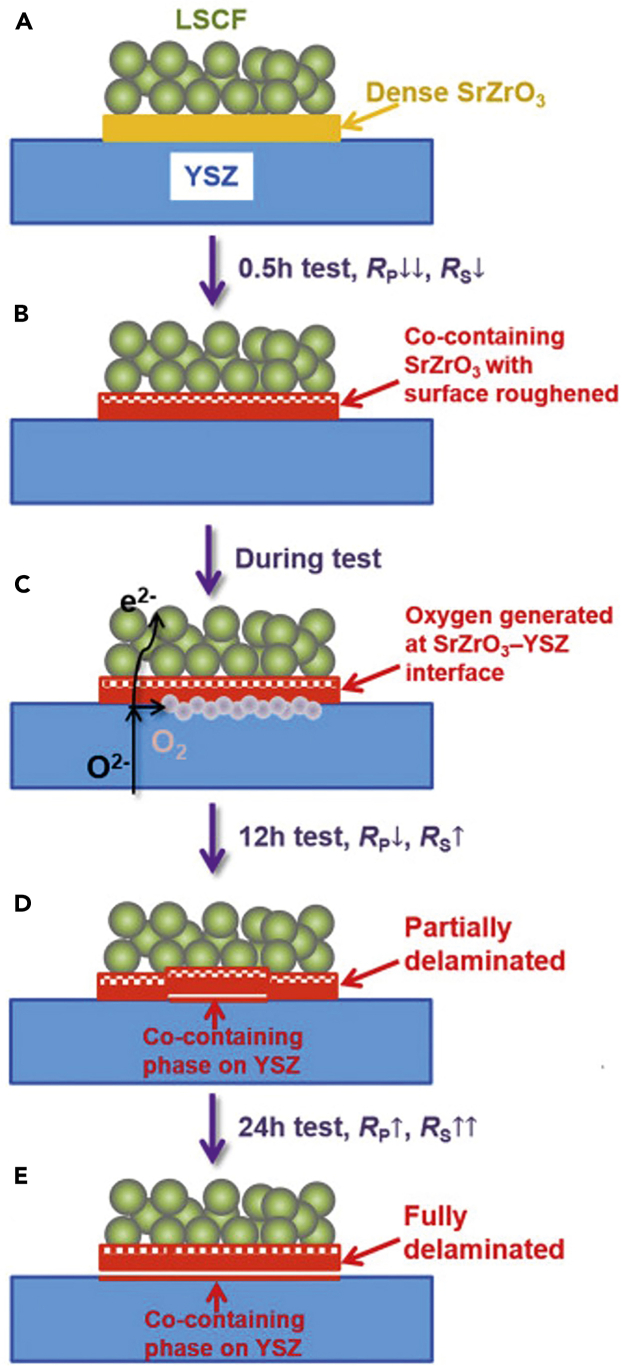
Mechanism for the degradation process Diagram showing the process that leads to the activation and subsequent detachment of the LSCF air electrode: (A) the initial, freshly prepared state of the sample; (B) the sample’s condition following half an hour of high-current electrolysis; (C) the sample’s status amidst the high-current electrolysis examination; (D) the sample’s appearance after 12 h of electrolysis testing; and (E) the final state of the sample after completing a full 24-h electrolysis test. Adapted from^154^ with permission; copyright 2018, Science Direct.

LSCF electrodes suffer from the diffusion of species like strontium (Sr) and cobalt (Co), leading to phase changes and loss of electrochemical activity.^155^^,^^157^ Delamination occurrences have been documented. For instance, Pan and colleagues [53] observed delamination of the LSCF anode in a scenario where a three-electrode cell was subjected to an anodic current of 1 A/cm^2^ at a temperature of 800°C for 24 h. The formation of SrZrO_3_ was observed, and a mechanism for the degradation was proposed, as illustrated in Figure 13. The scheme shows the stages of activation and delamination of an LSCF air electrode under electrolysis. It shows the initial condition of the fresh sample and its progression through 0.5, 12, and 24 h of high-current electrolysis testing. The sequence captures the changes in the electrode over time, culminating in delamination after extended electrolysis.^154^

Electrolyte degradation primarily concerns the stability of YSZ and GDC under SOECs' operating conditions. YSZ is vulnerable to grain boundary weakening and microstructural changes, particularly under the dual influences of thermal cycling and prolonged exposure to high steam and oxygen partial pressures.^158^^,^^159^^,^^160^ GDC, while exhibiting higher ionic conductivity than YSZ, can undergo reduction of Ce^4+^ to Ce^3+^, particularly under reducing conditions, which compromises its structural integrity and ionic conductivity.^161^ At the electrodes and electrolytes' interfaces, stability is largely governed by the oxygen partial pressure (pO_2_). The variation in pO_2_ across the cell components can lead to myriad degradation phenomena, including cation diffusion and the formation of secondary phases. These interfacial phenomena are crucial as they directly affect the mechanical integrity and ionic conductivity of the SOEC.^156^

Thus, the degradation mechanisms of SOECs involve a complex interplay of chemical reactions, material instability, and mechanical stresses, all exacerbated by the harsh operating conditions typical in SOEC applications. Possible mitigation processes include: (1) innovative infiltration techniques to enhance the durability and functionality of the electrode materials, e.g., introducing fine particles of catalysts into the electrode structure, which can help improve the performance and stability of the electrodes under operation^162^^,^^163^^,^^164^^,^^165^; (2) exploration and development of new anode materials more resistant to the harsh operating conditions of SOECs, such as high temperatures, mechanical stresses, and chemical reactions that typically lead to degradation^166^^,^^167^^,^^168^; and (3) designing the materials at the micro-level to optimize their properties, such as ionic and electronic conductivity, structural integrity, and reaction sites availability.^169^^,^^170^

## Prospects, research directions, and conclusions

Integrating SOECs with CH_4_ assistance and FT synthesis promises transformative advancements in sustainable fuel production. The potential of this technology extends beyond merely reducing carbon emissions; it aligns with global energy sustainability goals by turning greenhouse gases into valuable fuel resources. Emerging trends indicate a growing emphasis on improving industrial processes' energy efficiency and environmental impact. In addition, SOEC technology, particularly when integrated with renewable energy sources, represents a significant step toward achieving these goals. The use of CH_4_ in SOEC processes enhances the H_2_:CO ratio crucial for efficient FT synthesis and helps reduce the energy required for electrolysis, thereby improving overall system efficiency.

The approaching industrial applications of CH_4_-assisted SOEC systems are vast. These range from large-scale synthetic fuel production facilities, which can produce cleaner-burning alternatives to conventional fossil fuels, to chemical manufacturing processes that require syngas as a feedstock. Additionally, the flexibility of SOEC systems to adjust syngas compositions makes them suitable for producing specialized chemicals and fuels tailored to specific industrial needs. Despite its potential, the commercial adoption of SOEC technology faces several challenges. One of the primary limitations is the durability of the electrolysis cells, particularly at the high operating temperatures required for efficient operation. Research is urgently needed to develop more robust materials that can withstand these conditions without degrading. Moreover, the integration of CH_4_ and the optimization of syngas production require further exploration to prevent carbon deposition on the catalysts, which can lead to decreased efficiency and system failure.

The complex interplay of reactions within SOEC systems, especially when incorporating other molecules such as nitrogen oxides or sulfur-containing compounds, poses additional challenges. These molecules could provide pathways to a broader range of chemicals but require tailored catalysts and operating conditions to ensure efficient conversion and minimal environmental impact. Importantly, these analyses should cover experimental procedures and produce computational simulations and theoretical models to understand better the reaction mechanisms and materials’ behaviors under operational conditions.

Particularly, density functional theory (DFT) and density functional tight binding (DFTB) calculations are powerful tools for this purpose. These theoretical approaches can provide detailed insights into the electronic structures and potential energy surfaces of the materials and reactions involved in SOEC systems. By employing DFT/DFTB calculations, researchers can predict the interaction dynamics between various molecules and the catalyst surface, assess the stability of materials under different conditions, and optimize the designs of catalysts to prevent carbon deposition and enhance overall system efficiency. Such theoretical studies are crucial for identifying optimal operating parameters, designing materials with desired properties, and understanding electrolysis and synthesis processes’ complex kinetics and thermodynamics. These insights can significantly reduce the time and cost of experimental trials by pinpointing promising avenues of research and development. Combining theoretical calculations and empirical research will ultimately be essential to refine the technology, overcome current limitations, and achieve the scalable application of CH_4_-assisted SOEC and Fischer–Tropsch synthesis in sustainable fuel production.

### Limitations of the study

Due to space limitations, some important works may not be fully discussed and acknowledged.

## Acknowledgments

The authors gratefully acknowledge the financial support of Petrogal Brasil S.A., the Brazilian National Agency for Petroleum, Natural Gas and Biofuels (ANP), and 10.13039/501100003758Fundação de Amparo à Pesquisa e ao Desenvolvimento Científico e Tecnológico do Maranhão - FAPEMA.

## Author contributions

M.M., R.L.S.J., J.M.A.R.A., P.N.R., and M.A.S.G. are responsible for writing the article and have the equal contribution. M.A.S.G. made the drawings and adjustments to the references. J.M.A.R.A. and P.N.R. supervised the project.

## Declaration of interests

There are no conflicts of interest to disclose.
